# NF-κB1 Regulates Immune Environment and Outcome of Notch-Dependent T-Cell Acute Lymphoblastic Leukemia

**DOI:** 10.3389/fimmu.2020.00541

**Published:** 2020-04-03

**Authors:** Paola Grazioli, Andrea Orlando, Nike Giordano, Claudia Noce, Giovanna Peruzzi, Gaia Scafetta, Isabella Screpanti, Antonio Francesco Campese

**Affiliations:** ^1^Department of Experimental Medicine, Sapienza University, Rome, Italy; ^2^Department of Molecular Medicine, Sapienza University, Rome, Italy; ^3^Center for Life Nano Science@Sapienza, Istituto Italiano di Tecnologia, Rome, Italy; ^4^Department of Medico-Surgical Sciences and Biotechnologies, Sapienza University, Rome, Italy

**Keywords:** Notch, NF-κB1, T-ALL, Tregs, tumor environment, myeloproliferation

## Abstract

T-cell acute lymphoblastic leukemia (T-ALL) is an aggressive pediatric malignancy that arises from the transformation of immature T-cell progenitors and has no definitive cure. Notch signaling governs many steps of T cell development and its dysregulation represents the most common causative event in the pathogenesis of T-ALL. The activation of canonical NF-κB pathway has been described as a critical downstream mediator of Notch oncogenic functions, through the sustaining of tumor cell survival and growth. The potential role of Notch/NF-κB partnership is also emerging in the generation and function of regulatory T cells (Tregs) in the context of cancer. However, little is known about the effects of combined mutations of Notch and NF-κB in regulating immune-environment and progression of T-ALL. To shed light on the topics above we generated double-mutant mice, harboring conventional *knock-out* mutation of NF-κB1/p50 on the genetic background of a transgenic model of Notch-dependent T-ALL. The immunophenotyping of double-mutant mice demonstrates that NF-κB1 deletion inhibits the progression of T-ALL and strongly modifies immune-environment of the disease. Double-mutant mice display indeed a dramatic reduction of pre-leukemic CD4^+^CD8^+^ (DP) T cells and regulatory T cells (Tregs) and, concurrently, the rising of an aggressive myeloproliferative trait with a massive expansion of CD11b^+^Gr-1^+^ cells in the periphery, and an accumulation of the granulocyte/monocyte progenitors in the bone-marrow. Interestingly, double-mutant T cells are able to improve the growth of CD11b^+^Gr-1^+^ cells *in vitro*, and, more importantly, the *in vivo* depletion of T cells in double-mutant mice significantly reduces the expansion of myeloid compartment. Our results strongly suggest that the myeloproliferative trait observed in double-mutant mice may depend on non-cell-autonomous mechanism/s driven by T cells. Moreover, we demonstrate that the reduction of CD4^+^CD8^+^ (DP) T cells and Tregs in double-mutant mice relies on a significant enhancement of their apoptotic rate. In conclusion, double-mutant mice may represent a useful model to deepen the knowledge of the consequences on T-ALL immune-environment of modulating Notch/NF-κB relationships in tumor cells. More importantly, information derived from these studies may help in the refinement of multitarget therapies for the disease.

## Introduction

The canonical Notch signaling represents a highly conserved pathway starting with interaction between transmembrane receptors (Notch1–4, in mammals) and ligands (Jagged−1 and −2 and Dll−1,−3, and −4, in mammals), that are expressed on neighboring cells. This event leads to the release of a functional intracellular domain (ICN), that translocates into the nucleus, where it combines with the DNA-binding factor CSL/RBP-Jk and other co-factors and regulates transcription of numerous genes (1). Notch impinges on a plethora of transduction pathways, the combination of which produces pleiotropic effects and context dependent modulations of the signal (2). This variability enables Notch to regulate disparate processes in health and disease, including cancer, where it may act as an oncogene or, conversely, as a tumor suppressor (3). In the hematopoietic system, Notch receptors exert fundamental roles in multiple steps of T cell development and in differentiation of T cell subsets (4, 5), including immunosuppressive Foxp3^+^regulatory T cells (Tregs) [as reviewed in (6)]. Not surprisingly, deregulated activation of Notch1 or Notch3 causes the development of “T-cell acute lymphoblastic leukemia (T-ALL)”, in both mice and humans (7–11). T-ALL represents an aggressive pediatric malignancy that derives from the transformation of hematopoietic progenitors and has no definitive cure (3).

NF-κB is a large family of inducible transcription factors that controls important cellular processes, such as differentiation, survival, and proliferation. NF-κB canonical pathway starts with the activation of the IκB kinase (IKK) complex by different stimuli that leads to the degradation of IκB, the NF-κB inhibitor. Then, the NF-κB dimers, mainly represented by the association of RelA/p65 with NF-κB1/p50, are able to translocate into the nucleus and to activate many targets (12). NF-κB controls T cell development and differentiation at multiple stages (13) and represents a crucial downstream effector of Notch signaling in both physiological and pathological conditions. The functional relationship of Notch with NF-κB has been well established in T-ALL onset and progression (8, 14–18), in addition to the interaction of Notch with other important oncogenic mediators, such as pre-TCR/pTα and Ikaros (19–23). Recently, literature is pointing out the function of Notch in shaping tumor microenvironment (24). In particular, Notch is involved in the regulation of tumor immune response (25–28). However, details about the influence of Notch and/or NF-κB on the immune-environment of T-ALL are very limited (29–34). A possible role for Notch/NF-κB partnership has been also suggested in Tregs in the context of cancer (35). Notably, increased percentages of Tregs has been associated with poor prognosis in T-ALL patients (36). However, no studies as yet addressed the effects of Notch/NF-κB interaction on Tregs in T-ALL.

Previously, we demonstrated that *lck*-driven deregulation of Notch3-ICN inside T cell compartment of transgenic (*N3tg*) mice induces an aggressive disease with the features of juvenile T-ALL and characterized by the accumulation of pre-leukemic CD4^+^CD8^+^ (DP) T cells in the periphery (8). The expansion of tumor cells is sustained by the constitutive activation of the p65/p50 NF-κB complex (8). Moreover, T-ALL development in *N3tg* mice is associated to enhanced generation of “natural” Tregs (37). Importantly, deletion of the PKCθ kinase, which mediates activation of canonical NF-κB, reduces incidence of leukemia in *N3tg* mice (14). Finally, we also reported that Notch3, PKCθ, and p65/NF-κB co-operate in modulating Foxp3 transcription in Tregs (38).

However, how the deletion of NF-κB components may affect disease progression and Treg behavior in Notch-dependent T-ALL has not yet been investigated. To this end, we generated double-mutant mice, harboring NF-κB1/p50 deletion on a T-cell targeted Notch3-transgenic background. The characterization of this model suggests that inhibition of NF-κB1 delays the progression of T-ALL and modifies immune-environment of the disease, by inducing a dramatic reduction of DP T cells and Tregs and concurrently the rising of an aggressive T-cell dependent myeloproliferative trait.

## Materials and Methods

### Mice

We intercrossed *N3tg* (8) and *p50*^−/−^ (39) mice, both on C57BL/6 background, to generate *N3tg/p50*^−/−^ double-mutant mice, that were bred and housed under specific pathogen-free conditions. The *p50*^−/−^ mice were obtained from Jackson Laboratories, Bar Harbor, ME, USA. All mice were monitored daily and euthanized upon disease detection (8), as evidenced by enlarged spleen, hunched posture, ruffled fur, reduced mobility, and/or labored breathing. Experimental groups were based on age and genotype of mice. The Foxp3EGFP reporter mice (38), are “knock-in” mice on C57BL/6 background, overexpressing an IRES-EGFP cassette in the 3′ untranslated region of Foxp3 gene. The number of mice used in each experiment was reported in figure legends. Animal studies were approved by the local Animal welfare committee and were carried out in accordance with the recommendations of the Italian national guidelines for experimental animal care and use and of the European Directive 2010/63/EU.

### Cell Preparation and Flow Cytometry

Single-cell suspensions were prepared from thymus, spleen, bone-marrow, or peripheral blood in 1x PBS supplemented with 2% FBS and erythrocytes were lysed with ammonium-chloride-potassium lysing buffer, as previously reported (40). Freshly isolated cells were stained with surface markers for 30 min on ice using the following antibodies: CD4 (RM4-5), CD8a (53–6.7), CD11b (M1/70), Gr-1(RB6-8C5), all from BD Biosciences. For Treg detection cells were stained with the following surface antibodies: CD4 (RM4-5), CD8a (53-6.7), CD25 (PC61) (all from BD Biosciences) and then with the intracellular Foxp3 antibody (FJK-16s) (eBioscience), by using Foxp3/Transcription Factor Staining Buffer Set (eBioscience), following manufacturer's instructions. Samples above were run on a FacsCalibur (BD Biosciences) and analyzed with the CellQuest Pro software (BD Biosciences).

To evaluate early myeloid progenitors distribution, bone-marrow cells were stained with the APC mouse lineage Ab cocktail (BD Biosciences), and with the APC-CD4, APC-CD8a, and APC-IL7Ra antibodies (BD Biosciences), to determine the Lineage negative (Lin^−^) subset, and then with the APCH7-cKit (2B8), PerCPCy5.5-Sca-1(D7), FITC-CD34 (RAM34), and PE-FcgRIII/II (2.4G2) antibodies (BD Biosciences). Analysis of apoptosis was performed in gated CD4^+^CD8^+^ DP T subset by staining cells with surface markers V450-CD4 (RM4-5) and PE-CD8a (53–6.7), and then by labeling cells with BUV395-Annexin V and 7-AAD (BD Biosciences), as previously described (41). In order to evaluate apoptosis in Tregs, cells were stained with surface markers PerCPCy5.5-CD4 (RM4-5) and FITC-CD8a (53–6.7), then labeled with Fixable Viability Stain 780 and APC-Annexin V (both from BD Biosciences), just prior to fixation/permeabilization and staining with PE-Foxp3 (FJK-16s), performed as above. Proliferation of CD4^+^CD8^+^ DP T cells was assessed by intracellular staining with the BV510-Ki-67 (B56) antibody (BD Biosciences). For cell cycle analysis 7AAD (Sigma) was used at 25 mg/ml with RNase (Sigma) 40 mg/ml. All intracellular stainings were performed with Foxp3/Transcription Factor Staining Buffer Set (eBioscience). For the intracellular staining with anti-pSTAT5/pY694 (47/STAT5; BD Bioscience) T splenocytes were isolated by using Pan T cell isolation kit (Miltenyi), following manufacturer's instructions. Before the staining, the cells were either left unstimulated or stimulated with increasing doses of rhIL2 (Peprotech) for 15 min at 37°C and then, were fixed and permeabilized with Transcription Factor Phospho Buffer Set (BD Bioscience) according to the manufacturer's instructions. Samples were run on BD LSRFortessa equipped with DIVA software (BD Biosciences) and data were elaborated using FlowJo software (TreeStar).

### *In vivo* T-Cell Depletion

*N3tg/p50*^−/−^ double-mutant mice at 3 weeks of age were injected intraperitoneally with 250 μg of InVivoPlus anti-mouse CD8α (2.43 clone), plus 250 μg of InVivoPlus anti-mouse CD4 (GK1.5 clone) or with 500 μg of InvivoPlus RatIgG2b (LTF-2 clone) isotype control (all from BioXCell), resuspended in 200 μl/mouse of PBS 1x, twice a week. After 3 weeks of treatment mice were sacrificed and characterized by FACS analysis, as described above. In particular, to evaluate the distribution of T cell subsets we used the following surface antibodies: CD4 (RM4-4, BD Bioscience) and CD8β (H35-17.2, eBioscience).

### Cell Sorting

For sorting experiments, bone-marrow samples were obtained from *wt, N3tg*, and *N3tg/p50*^−/−^ mice, as above. Mononuclear cells were isolated by ficoll gradient and stained with a “lineage cocktail” containing PE-conjugated monoclonal antibodies against CD11b, Gr-1, Ter119, CD45R, CD3ϵ, CD4, and CD8a. “Lineage negative” (Lin^−^) cells were purified with the FACSAria cell sorter (BD Biosciences; purity ≥ 98%).

In other sets of experiments, thymus or spleen cell suspensions were obtained and stained with anti-CD4 and anti-CD8 surface markers, as above. Then CD4^+^CD8^+^ (DP), CD4^+^CD8^−^, or CD4^−^CD8^+^ T cells from *wt, N3tg*, and *N3tg/p50*^−/−^ mice, or CD4^+^CD8^−^EGFP^+^ Tregs from spleen of Foxp3EGFP reporter mice (see above), were isolated (purity ≥ 98%) using a BD FACSAria III (BD Biosciences), equipped with FACSDiva software (BD Biosciences), as previously described (42).

### Colony Forming Unit Assay

For myeloid colony forming unit assay, Lin^−^ cells (5 × 10^3^ cells/well), purified as above, were plated in triplicate in methylcellulose-based semisolid medium (Methocult M3236, Stem Cell Technology) with FCS and the following cytokines: IL-3 (2 ng/ml), IL-6 (2 ng/ml), SCF (50 ng/ml), G-CSF (50 ng/ml), and GM-CSF (10 ng/ml). After 7–10 days colonies were analyzed and counted and cells were harvested, controlled for the expression of myeloid markers by FACS analysis and then replated (10 × 10^3^/well) to assess survival.

### Cell Culture

All the cell culture samples were cultured at 37°C and 5% CO_2_ in complete medium, that is RPMI-1640 medium (GIBCO), supplemented with 10% FBS, 10 U/ml penicillin and streptomycin, 2 mM glutamine. In particular, total cells obtained from bone-marrow of *wt* mice (0.25 × 10^6^/well) were co-cultured 1:1 in 96 well plates with total T splenocytes from *wt, N3tg*, or *N3tg/p50*^−/−^ mice. T splenocytes were isolated by using Pan T cell isolation kit (Miltenyi), following manufacturer's instructions. After 24 and 48 h of co-culture, cells were counted and stained with CD11b and Gr-1 antibodies for FACS analysis, as above. In some experiments, the co-culture assay was conducted by using CD4^+^CD8^+^ (DP), CD4^+^CD8^−^, or CD4^−^CD8^+^ T splenocytes sorted from double-mutant mice at purity ≥ 98%, as described previously. To check for the role of cell-to-cell contact mechanisms, we performed the co-culture experiments above in 96 well plates in the presence of transwell inserts (pore diameter, 0.4 μm, Corning). Finally, for testing the possible role of Tregs, total cells obtained from bone-marrow of *wt* mice (0.25 × 10^6^/well) were co-cultured 1:1 in 96 well plates with total T splenocytes from *N3tg/p50*^−/−^ mice, without or with different numbers of CD4^+^CD8^−^EGFP^+^ Tregs (1 × 10^4^/3 × 10^4^/6 × 10^4^), purified from spleen of Foxp3EGFP reporter mice (see above).

In another set of experiments, freshly isolated thymocytes (0.5 × 10^6^/well) were cultured in 96 well plates in the following conditions: untreated, upon TCR activation with coated anti-CD3 antibodies (145-2C11) 3 μg/ml, or in the presence of rhIL2 (Peprotech) 50 U/ml or murine IL-15 (Peprotech) 50 ng/ml. Then, apoptosis was assessed at 48 h by FACS analysis, as described above.

Finally, T splenocytes from *wt, N3tg*, or *N3tg/p50*^−/−^ mice (0.5 × 10^6^/well), purified as above, were cultured in 96 well plates and stimulated or not with coated anti-CD3 antibodies (145-2C11) 3 μg/ml plus rhIL2 (Peprotech) 50 U/ml, in triplicates. Then, at 24 and 48 h, cells were counted and stained to assess Treg numbers by flow cytometry, as described above.

### mRNA Analysis

CD4^+^CD8^+^ thymocytes purified as above were processed to extract total RNA with TRIzol reagent (Invitrogen), and reverse transcription was performed with High-Capacity cDNA Reverse Transcription Kit (ThermoFisher), according to the manufacturer's protocol. TaqMan quantitative real-time PCR (qPCR) was performed on cDNA using the StepOnePlus™ Real-Time PCRSystem (ThermoFisher), following instructions of manufacturer. Taqman Gene Expression Master Mix and Taqman Gene Expression Assays on demand for Bcl2 (Mm00477631_m1), A1 (Mm03646861_mH), and Hprt (Mm01545399_m1) were purchased from ThermoFisher. Relative quantification was carried out using the comparative ΔΔ*C*t method. Hprt expression was used to normalize the expression levels of mRNAs.

### Western Blotting

Total protein extracts were prepared from sorted DP T thymocyte samples and Western blotting analysis was conducted, as previously described (43), using anti-p21 antibody (C-19, sc-397, Santa Cruz), and anti-Caspase-3 or anti-cleaved Caspase-3 antibody (#9665 and #9661, respectively, both from Cell signaling). Anti-β-actin antibody (Sigma-Aldrich), was used to normalize protein expression levels. Densitometric analysis was performed using ImageStudio software (LI-COR Biosciences).

### Statistics

Results were expressed as means ± SD. We performed unpaired two-tailed Student's test. Differences were considered significant when ^*^P ≤ 0.05, ^**^P ≤ 0.01, ^***^P ≤ 0.001, and ^****^P ≤ 0.0001. Kaplan-Meier survival analysis was performed comparing kinetics of disease development in *N3tg/p50*^−/−^, *N3tg, wt*, and *p50*^−/−^ mice. *P*-value was calculated by Log rank (Mantel-Cox) test. Statistical analysis was performed using Prism, GraphPad software.

## Results

### Deletion of NF-κB1/p50 Delays Progression of Notch3-Dependent T-ALL and Induces Myeloproliferation

**Figure 1:**
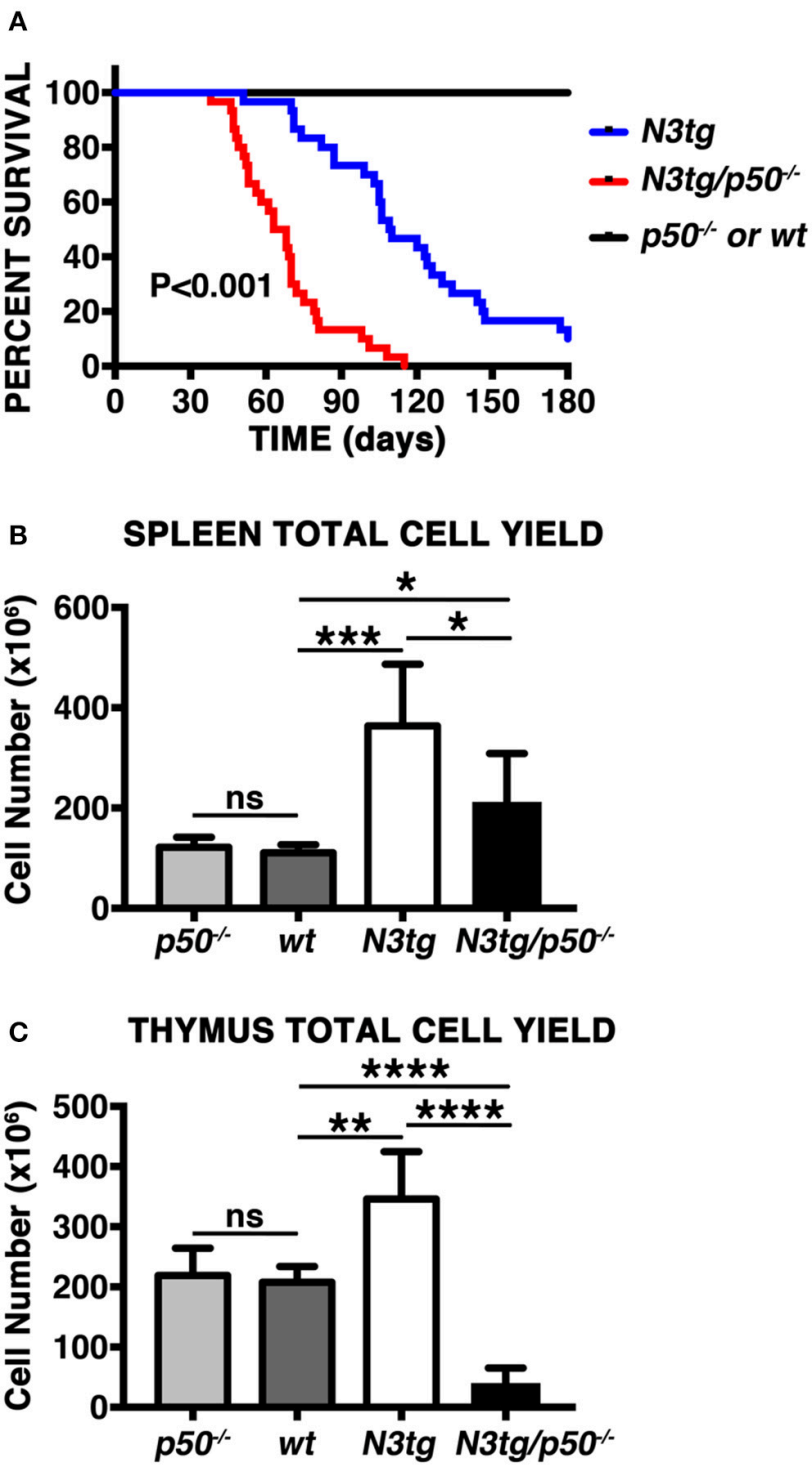
NF-κB1 deletion modifies T-ALL features in *N3tg/p50*^−/−^ vs. *N3tg* mice. **(A)** Kaplan-Meier survival plot showing disease latency in *N3tg/p50*^−/−^ (*n* = 30), *N3tg* (*n* = 30), *p50*^−/−^ (*n* = 15), and *wt* (*n* = 15) mice. *P*-value was calculated by Log rank (Mantel-Cox) test. **(B)** Total cell counts of the spleen from *N3tg/p50*^−/−^, *N3tg, p50*^−/−^, and *wt* mice at 8–9 weeks of age. **(C)** Total cell counts of the thymus from *N3tg/p50*^−/−^, *N3tg, p50*^−/−^, and *wt* mice at 4–5 weeks of age. In **(B,C)** the values are presented as mean ± SD of at least five independent experiments (*n* ≥ 5 mice per group). ns, not significant; *P ≤ 0.05, **P ≤ 0.01, ***P ≤ 0.001, and ****P ≤ 0.0001 represent significant differences between the indicated groups.

In order to analyze the effects of canonical NF-κB pathway modulation on Notch3-dependent T-ALL we generated *N3tg/p50*^−/−^ double-mutant mice, by intercrossing NF-κB1/p50^−/−^ mice (39), with *N3tg* animals (8). Surprisingly, the follow-up of *N3tg/p50*^−/−^, *N3tg*, and relative control mice (*wt* and *p50*^−/−^), revealed that *N3tg/p50*^−/−^ double-mutant mice had a median survival of 65.5 days; in contrast, *N3tg* mice showed a median life span of 109.5 days (Figure 1A). Notably, *N3tg/p50*^−/−^ mice presented clinical signs that were different from typical features of T-ALL, routinely observed in *N3tg* animals (8). At the end point, *N3tg/p50*^−/−^ mice appeared indeed smaller in size with respect to *wt* or single mutant controls (not shown). Moreover, disease of *N3tg/p50*^−/−^ mice at 8–9 weeks of age was accompanied by splenomegaly, though less pronounced than that observed in *N3tg* mice (Figure 1B and not shown). Finally, the thymus of double-mutant mice was dramatically reduced in size (Figure 1C and not shown), starting at 4–5 weeks of age.

**Figure 2:**
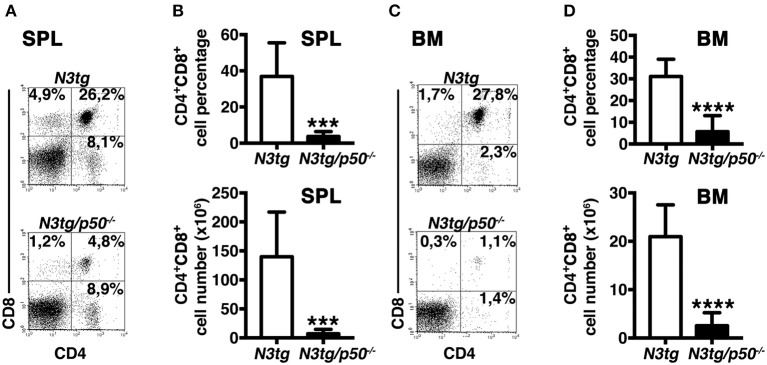
Reduced expansion of CD4^+^CD8^+^ (DP) T cells in *N3tg/p50*^−/−^ mice. Representative dot plots showing CD4 vs. CD8 distributions in the spleen **(A)** and BM **(C)** of *N3tg/p50*^−/−^ vs. *N3tg* mice at 8–9 weeks of age, as assessed by flow cytometry. Numbers inside each cytogram represent the percentages of different cell subsets. **(B)** Percentages and absolute numbers of DP T cells in the spleen (SPL) of *N3tg/p50*^−/−^ vs. *N3tg* mice, measured as in **(A)**. **(D)** Percentages and absolute numbers of DP T cells in the bone marrow (BM) of *N3tg/p50*^−/−^ vs. *N3tg mice*, measured as in **(C)**. In **(B,D)** the values are presented as mean ± SD of five independent experiments (*n* ≥ 5 mice per group). ***P ≤ 0.001 and ****P ≤ 0.0001 represent significant differences between the indicated groups.

**Figure 3:**
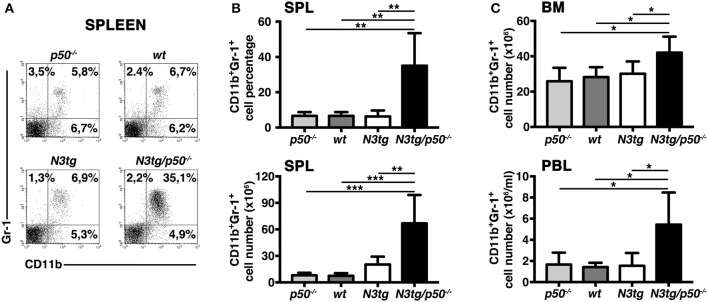
Enlargement of CD11b^+^Gr-1^+^ subset in *N3tg/p50*^−/−^ mice. **(A)** Representative dot plots showing CD11b vs. Gr-1 distributions in the spleen of *N3tg/p50*^−/−^, *N3tg, p50*^−/−^, and *wt* mice at 8–9 weeks of age, as measured by FACS analysis. Numbers inside each cytogram represent the percentages of different cell subsets. **(B)** Percentages (*upper panel*) and absolute numbers (*lower panel*) of CD11b^+^Gr-1^+^ cells in the spleen from *N3tg/p50*^−/−^, *N3tg, p50*^−/−^, and *wt* mice, measured as in **(A)**. **(C)** absolute numbers of CD11b^+^Gr-1^+^ cells in the BM (*upper panel*) and peripheral blood lymphocytes (PBL) (*lower panel*) from *N3tg/p50*^−/−^, *N3tg, p50*^−/−^, and *wt* mice, measured as in **(A)**. In **(B,C)** data represent mean ± SD of five independent experiments (*n* ≥ 5 mice per group). *P ≤ 0.05, **P ≤ 0.01, and ***P ≤ 0.001 represent significant differences between the indicated groups.

To clarify the nature of double-mutant mice pathology we performed immunophenotypic analysis of hematopoietic cell subsets in different organs from *N3tg/p50*^−/−^, *N3tg, p50*^−/−^, and *wt* mice, by FACS analysis. Regarding the T cell compartment, we focused on immature CD4^+^CD8^+^ (DP) T-cell population. These cells are normally confined to the thymus, while their presence in the periphery represents a reliable marker to follow T-ALL progression (44–46). CD4^+^CD8^+^ (DP) T cells were highly decreased in percentages and numbers in both spleen (SPL; Figures 2A,B) and bone-marrow (BM; Figures 2C,D) of *N3tg/p50*^−/−^ vs. *N3tg* mice at 8–9 weeks of age, whereas they were virtually absent in organs from *p50*^−/−^ and *wt* controls (not shown). Conversely, the analysis of myeloid cell distributions revealed marked expansion of CD11b^+^Gr-1^+^cells in the spleen (Figures 3A,B), as well as in the BM (Figure 3C, *upper panel*) and peripheral blood (Figure 3C, *lower panel*) of *N3tg/p50*^−/−^ mice at 8–9 weeks of age, when compared with *N3tg, p50*^−/−^, and *wt* counterparts. Collectively, our results indicate that the deletion of NF-κB1 in *N3tg* mice induces a delay of T-ALL progression on one hand, and promotes myeloproliferation on the other hand, thus affecting the composition of T-ALL immune-environment.

### Expansion of “Granulocyte/Monocyte Progenitor”(GMP) Subset in the Bone-Marrow of *N3tg/p50^−/−^* Mice

**Figure 4:**
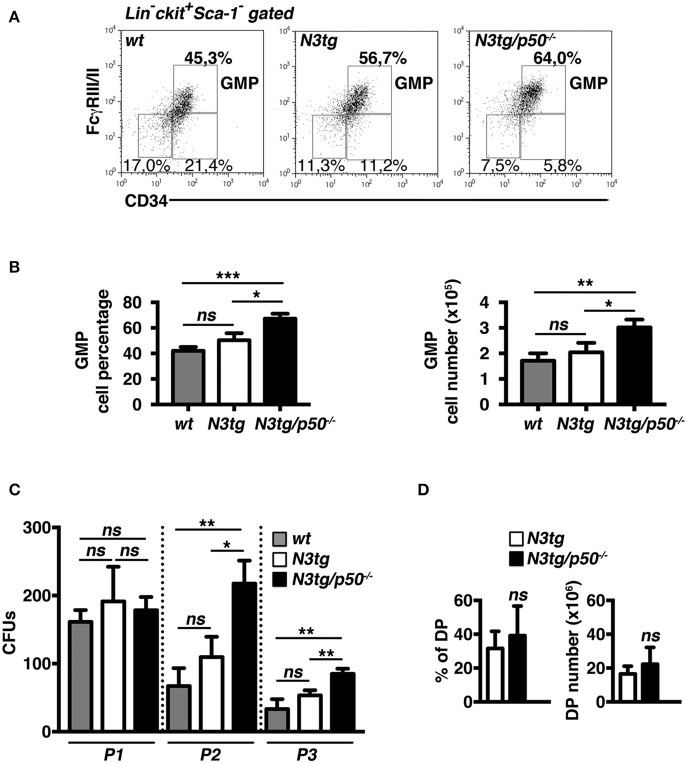
GMP progenitors accumulate in the BM of *N3tg/p50*^−/−^ mice. **(A)** Representative dot plots showing CD34 vs. FcγRIII/II distribution inside Lin^−^cKit^+^Sca-1^−^ gated cells in the BM of *N3tg/p50*^−/−^, *N3tg*, and *wt* mice at 4–5 weeks of age, as assessed by flow cytometry analysis of bone-marrow progenitor subsets (GMP, Granulocyte/Monocyte progenitors: CD34^+^FcγRII/III^+^; CMP, Common Myeloid progenitors: CD34^+^FcγRII/III^low^; MEP, Megacariocyte/Erithroid progenitors: CD34^−^FcγRII/III^low^), Numbers inside each cytogram represent the percentages of different subsets. **(B)** Percentages (*left panel*) and absolute numbers (*right panel*) of GMPs in the BM of *N3tg/p50*^−/−^, *N3tg* and *wt* mice, measured as in **(A)**. **(C)** Colony forming assay of purified BM Lin^−^ cells plated on semi-solid methylcellulose medium in the presence of factors stimulating the differentiation of progenitors toward a myeloid fate. Graph represents the mean number of colonies per plate (CFUs) obtained at any of the three re-plating points examined (P1, P2, and P3), for *N3tg/p50*^−/−^, *N3tg*, or *wt* cells. In **(B,C)** data represent mean ± SD of three independent experiments (*n* = 3 mice per group). ns, not significant; *P ≤ 0.05, **P ≤ 0.01, and ***P ≤ 0.001 represent significant differences between the indicated groups. **(D)** Percentages (*left panel*) and absolute numbers (*right panel*) of DP T cells in the BM of *N3tg/p50*^−/−^ vs. *N3tg mice* at 4–5 weeks of age, as measured by flow cytometry analysis of CD4 vs. CD8 distributions. The values are presented as mean ± SD of five independent experiments (*n* = 5 mice per group). ns, not significant differences between the groups.

The accumulation of CD11b^+^Gr-1^+^ cells suggested the presence of alterations in the myeloid cell development of *N3tg/p50*^−/−^ double-mutant mice. Thus, we analyzed the distribution of myeloid progenitor subsets [as defined by the differential expression of CD34 and FcγRII/III markers inside the Lin^−^ckit^+^Sca1^−^ compartment, see (47) and Figure 4A legend], in the bone-marrow of mice of different genotypes at 4–5 weeks of age. We revealed that double-mutant mice displayed a significant increase in the percentages (Figures 4A,B, *left panel*), and absolute numbers (Figure 4B, *right panel*) of “granulocyte/monocyte progenitor” **(**GMP) subset with respect to both *N3tg* and *wt* littermates, whereas the progenitor distribution in the BM of *p50*^−/−^ mice was similar to *wt* controls [(48) and not shown]. To confirm these data, we performed a myeloid “colony forming unit” assay with Lineage negative (Lin^−^) cells, purified from the bone-marrow of *N3tg/p50*^−/−^, *N3tg*, and *wt* mice, and plated on semi-solid methylcellulose medium in the presence of factors stimulating the differentiation of progenitors toward a myeloid fate (see the Materials and Methods section). As depicted in Figure 4C, the number of colonies/plate (CFUs) obtained at the P2 and P3 re-plating points, was significantly higher in *N3tg/p50*^−/−^ when compared to *N3tg* and *wt* mice.

Interestingly, immature CD4^+^CD8^+^ (DP) T cells are still largely represented in the BM of double-mutant mice at 4–5 weeks of age (Figure 4D), thus leaving open the possibility that these immature *N3tg/p50*^−/−^DP T cells may participate in inducing *in trans* the alteration of myeloid cell development described above.

### T Cells Sustain the Expansion of Myeloid Compartment in *N3tg/p50*^−/−^ Mice

**Figure 5:**
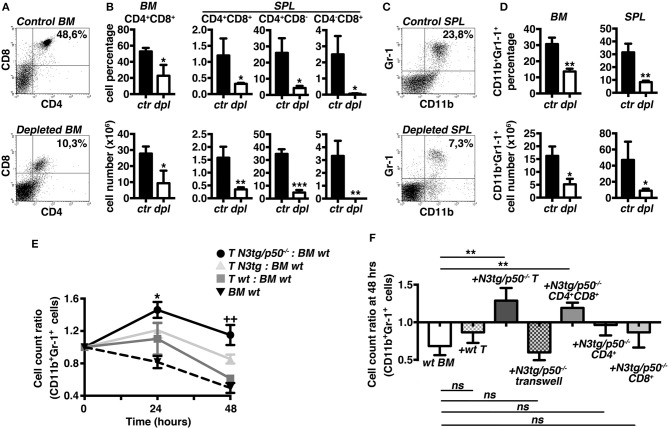
T cell compartment drives the expansion of myeloid cells in *N3tg/p50*^−/−^ mice. Three weeks-old *N3tg/p50*^−/−^ mice were injected i.p. with anti-CD4 plus anti-CD8 depleting antibodies or with relative isotype controls, bi-weekly for 3 weeks. **(A)** Representative dot plots showing CD4 vs. CD8 distributions in the BM of control (*upper panel*) or T-cell depleted (*lower panel*) *N3tg/p50*^−/−^ mice, as measured by FACS analysis. Numbers inside each cytogram represent the percentages of DP T cells. **(B)** Percentages (*upper panels*) and absolute numbers (*lower panels*) of BM CD4^+^CD8^+^ (DP) T cells and of (DP) T, CD4^+^CD8,^−^ and CD4^−^CD8^+^ splenocytes from control vs. T-cell depleted *N3tg/p50*^−/−^ mice (*ctr* and *dpl*, respectively), measured as in **(A)**. **(C)** Representative dot plots showing CD11b vs. Gr-1 distributions in the SPL of control (*upper panel*) or T-cell depleted (*lower panel*) *N3tg/p50*^−/−^ mice, as measured by FACS analysis. Numbers inside each cytogram represent the percentages of CD11b^+^Gr-1^+^ cells. **(D)** Percentages (*upper panels*) and absolute numbers (*lower panels*) of CD11b^+^Gr1^+^ cells in the BM and spleen from control vs. T-cell depleted *N3tg/p50*^−/−^ mice (*ctr* and *dpl*, respectively), measured as in **(C)**. In **(B,D)** data represent mean ± SD of three independent experiments (*n* = 3 mice per group). *P ≤ 0.05, **P ≤ 0.01, and ***P ≤ 0.001 represent significant differences between the T-cell depleted samples (*dpl*) and the relative controls (*ctr*) **(E)** The graph represents the ratio of the CD11b^+^Gr1^+^ cell count at 24 or 48 h to the related CD11b^+^Gr1^+^ cell count at 0 h in the co-cultures of *wt* BM cells with T splenocytes from *N3tg/p50*^−/−^, *N3tg* or *wt* mice and in the *wt* BM cells cultured alone, as a basal control. Data represent mean ± SD of three independent experiments (*n* = 3 mice per group), each in triplicates. *P ≤ 0.05 and ^**++**^*P* ≤ 0.01represent significant differences, at 24 and 48 h, respectively, between the values observed in co-cultures of double-mutant T splenocytes with *wt* BM cells (*T N3tg/p50*^−/−^*: BM wt*) and the values observed in the other co-culture samples (i.e., *T N3tg: BM wt*; *T wt: BM wt*; *BM wt* alone, as a basal control) **(F)**. The graph represents the ratio of the CD11b^+^Gr1^+^ cell count at 48 h to the related CD11b^+^Gr1^+^ cell count at 0 h in *wt* BM cells cultured alone (*wt BM*), in the co-cultures of *wt* BM cells with T splenocytes from *wt* mice (+*wt T*), or with T splenocytes from *N3tg/p50*^−/−^ mice in the absence or presence of transwell inserts (+*N3tg/p50*^−/−^*T* and + *N3tg/p50*^−/−^*T transwell*, respectively), and finally, in the co-cultures of *wt* BM cells with (DP) T, CD4^+^CD8^−^ or CD4^−^CD8^+^ T splenocytes purified from double-mutant mice *(*+*N3tg/p50*^−/−^*CD4*^+^*CD8*^+^, +*N3tg/p50*^−/−^*CD4*^+^, and +*N3tg/p50*^−/−^*CD8*^+^, respectively). Data represent mean ± SD of three independent experiments (*n* = 3 mice per group), each in triplicates. ns, not significant; **P ≤ 0.01 represents significant differences between the indicated groups. In **(E,F)** all the cells were from mice at 4–5 weeks of age.

To test the hypothesis that T cells may influence the myeloproliferation observed in double-mutant mice, we designed experiments of *in vivo* T-cell depletion, by the combined administration of anti-CD4 and anti-CD8 antibodies to *N3tg/p50*^−/−^ mice, starting at 3 weeks of age. After 3 weeks, *N3tg/p50*^−/−^ treated mice displayed a significant reduction of percentages (Figures 5A,B, *upper panels*) and absolute numbers (Figure 5B, *lower panels*) of all the examined T cell subsets in the BM and spleen, with respect to the *N3tg/p50*^−/−^ controls, as expected. Notably, T-cell depleted mice were characterized by a remarkable decrease in the proportion (Figures 5C,D, *upper panels*) and absolute numbers (Figure 5D, *lower panels*) of the CD11b^+^Gr-1^+^ subset in the same organs as above, when compared to the controls. Our results strongly suggest that T cells participate in sustaining the myeloid proliferation of double-mutant mice.

To gain more insight into the T/myeloid cell interaction in *N3tg/p50*^−/−^ mice, we performed *in vitro* co-culture experiments of total *wt* BM cells with T cells purified from the spleen of double-mutant, *N3tg* or *wt* mice at 4–5 weeks of age. Strikingly, we observed a significantly higher CD11b^+^Gr-1^+^ cell count ratio in the co-cultures with *N3tg/p50*^−/−^ T cells compared to the co-cultures with *N3tg* or *wt* T cells, at any time point considered (Figure 5E), indicating that *N3tg/p50*^−/−^ T cells may sustain the growth of CD11b^+^Gr-1^+^ cells *in vitro*. Then, we repeated the co-culture assay by using transwell inserts or by using different subsets of double-mutant T splenocytes, instead of total T splenocytes (Figure 5F). The results indicated that transwell inserts strongly inhibited the effect of *N3tg/p50*^−/−^ T cells on CD11b^+^Gr-1^+^ cell growth, thus suggesting that it requires cell-to-cell contact. Furthermore, we revealed that only CD4^+^CD8^+^ (DP) T cells from double-mutant mice are able to drive the expansion of myeloid compartment, whereas CD4^+^CD8^−^ and CD4^−^CD8^+^ T subsets do not influence this process, significantly. Overall, our *in vivo* and *in vitro* combined approaches suggest that the myeloproliferative trait of *N3tg/p50*^−/−^ mice may rely on a mechanism exerted *in trans* by *N3tg/p50*^−/−^ DP T cells and that requires cell-to-cell contact.

### Profound Alterations of T Cell Compartment in the Thymus and Spleen of *N3tg/p50*^−/−^ Mice

The thymus represents the natural environment in which DP T cell develop under the control of many pathways, including Notch and NF-κB. The reduction of DP T cell subset in the spleen and BM of double-mutant mice, coupled with thymus regression, suggested that such phenomena may rely on alterations of T cell development. This prompted us to analyze T cell compartment in more details. However, to minimize interference by T-ALL or myeloproliferation, we performed our studies in young mice, at 4–5 weeks of age, when *N3tg/p50*^−/−^ mice presented no evident symptoms of illness.

**Figure 6:**
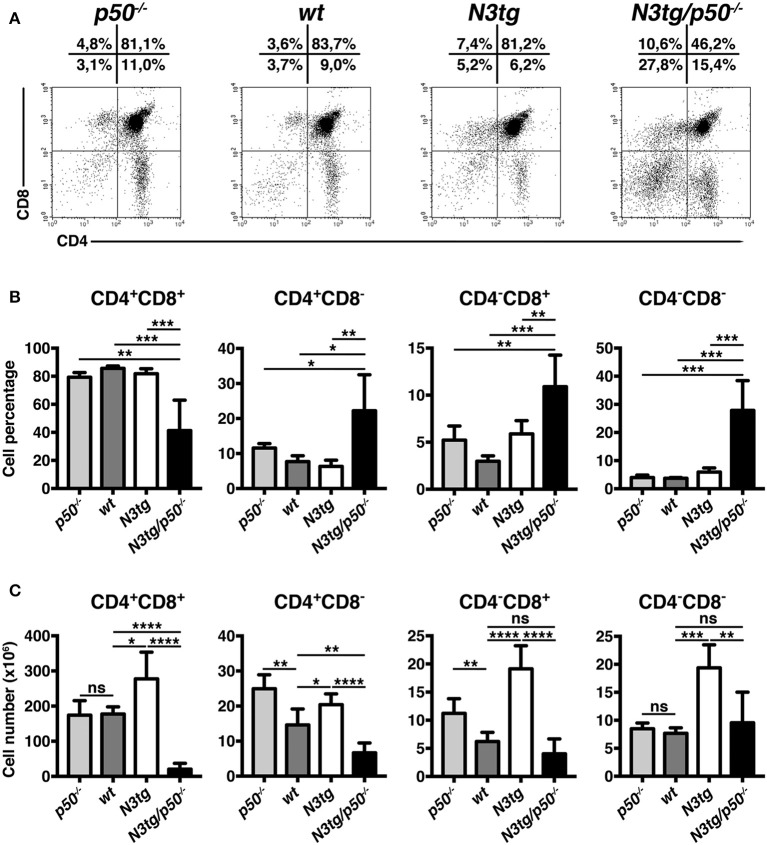
*N3tg/p50*^−/−^ mice display altered distributions of thymocyte subsets. **(A)** Representative dot plots showing CD4 vs. CD8 distributions in the thymus of *N3tg/p50*^−/−^, *N3tg, p50*^−/−^, and *wt* mice at 4–5 weeks of age, as measured by FACS analysis of CD4 vs. CD8 distributions. Numbers over each cytogram represent the percentages of different T cell subsets. **(B)** Percentages and **(C)** absolute numbers of thymocyte subsets in *N3tg/p50*^−/−^, *N3tg, p50*^−/−^, and *wt* mice, measured as in **(A)**. In **(B,C)** the values are presented as mean ± SD of five independent experiments (*n* ≥ 5 mice per group). ns, not significant; *P ≤ 0.05, **P ≤ 0.01, ***P ≤ 0.001, and ****P ≤ 0.0001 represent significant differences between the indicated groups.

Percentages (Figures 6A,B) and absolute numbers (Figure 6C) of CD4^+^CD8^+^ (DP) T cells were remarkably reduced in the thymus of *N3tg/p50*^−/−^ mice, when compared with those of *N3tg, p50*^−/−^ and *wt* mice. Concurrently, double-mutant mice presented increased percentages of CD4^+^CD8^−^, CD4^−^CD8^+^, and CD4^−^CD8^−^ thymocytes (Figures 6A,B). However, in terms of absolute numbers, the reduction observed in *N3tg/p50*^−/−^ vs. *N3tg* mice was not limited to DP T cells, but concerned also other subsets, with particular regard to CD4^+^CD8^−^ T cells (Figure 6C). Interestingly, the expansion of all thymocyte subsets that characterize *N3tg* vs. *wt* mice [(8) and Figure 6C], was abrogated in double-mutant mice. Moreover, the effects of NF-κB1 deletion on thymocytes emerged specifically in the Notch3 transgenic background, being absent in *p50*^−/−^ mice, when compared to *wt* controls. In summary, our results suggest that deletion of NF-κB1 may revert the consequences of Notch deregulated activation inside the thymic T-cell compartment.

**Figure 7:**
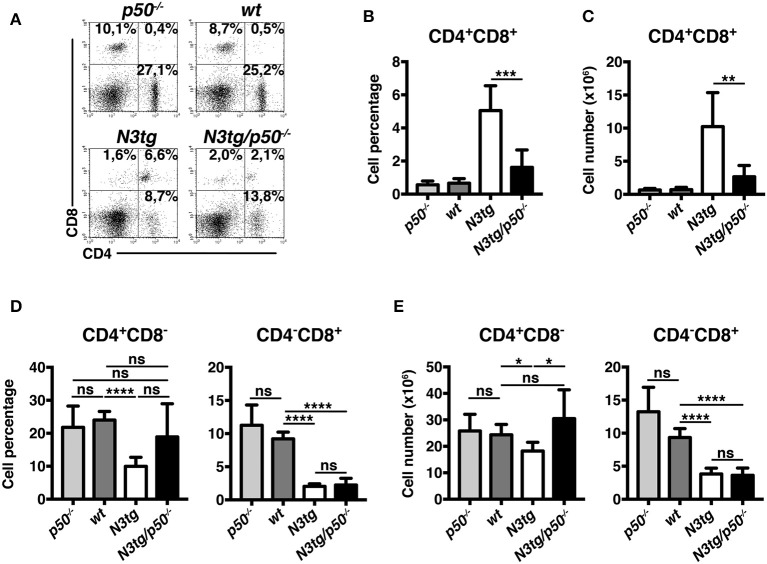
NF-κB1 deletion decreases CD4^+^CD8^+^ (DP) T cell numbers in the spleen of *N3tg/p50*^−/−^ vs. *N3tg mice*. **(A,B)** Percentages and **(C)** absolute numbers of DP T cells in the spleen of *N3tg/p50*^−/−^, *N3tg, p50*^−/−^, and *wt* mice at 4–5 weeks of age, as measured by FACS analysis of CD4 vs. CD8 distributions. In **(A)** numbers inside each cytogram represent the percentages of different cell subsets. **(D)** Percentages and **(E)** absolute numbers of CD4^+^CD8^−^ and CD4^−^CD8^+^subsets in the spleen of *N3tg/p50*^−/−^, *N3tg, p50*^−/−^ and *wt* mice, measured as in **(A)**. In **(B–E)** results are shown as mean ± SD of five independent experiments (*n* = 6 mice per group). ns=not significant; *P ≤ 0.05, **P ≤ 0.01, ***P ≤ 0.001 and ****P ≤ 0.0001 represent significant differences between the indicated groups.

Similar conclusions derived from analysis of splenic DP T cell compartment, that appeared significantly restricted in *N3tg/p50*^−/−^ vs. *N3tg* mice, in both percentages (Figures 7A,B) and absolute numbers (Figure 7C). These results suggest that the delay of T-ALL progression observed in *N3tg/p50*^−/−^ vs. *N3tg* mice at 8–9 weeks of age (see Figure 2), is already effective at an initial stage of the disease. We noted a slight decrease of CD4^+^CD8^−^ T cell percentages and absolute numbers (Figures 7D,E, respectively, *left panels*), in *N3tg* mice, with respect to those of *p50*^−/−^ and *wt* controls, though these reductions were recovered in double-mutant animals. Furthermore, percentages and absolute numbers of CD4^−^CD8^+^ T subset (Figures 7D,E, respectively, *right panels*), were reduced in *N3tg/p50*^−/−^ and *N3tg* mice in a similar way.

### Thymic and Splenic Treg Numbers Are Greatly Reduced in *N3tg/p50^−/−^* Mice

**Figure 8:**
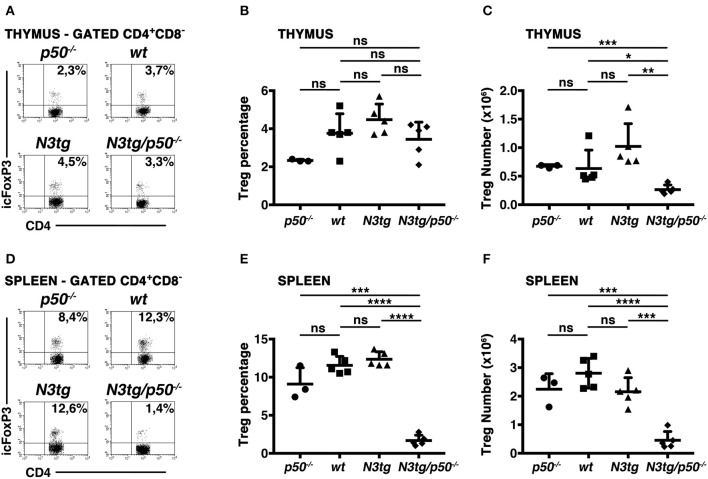
Tregs are reduced in the thymus and spleen of *N3tg/p50*^−/−^ double-mutant mice. **(A)** and **(D)** Representative FACS analysis of CD4^+^CD8^−^Foxp3^+^ Tregs in the thymus and spleen, respectively, of *N3tg/p50*^−/−^, *N3tg, p50*^−/−^ and *wt* mice at 4–5 weeks of age. Numbers inside each cytogram represent the percentages of Foxp3^+^ cells inside gated CD4^+^CD8^−^ cells. **(B)** and **(C)** Mean percentage values and absolute numbers, respectively, of CD4^+^CD8^−^Foxp3^+^ Tregs in the thymus from *N3tg/p50*^−/−^, *N3tg, p50*^−/−^, and *wt* mice, assessed as in **(A)**. **(E,F)** Percentages and absolute numbers, respectively, of CD4^+^CD8^−^Foxp3^+^ Tregs in the spleen from *N3tg/p50*^−/−^, *N3tg, p50*^−/−^, and *wt* mice, assessed as in **(D)**. In **(B,C,E,F)** the values are presented as mean ± SD of three independent experiments with *N3tg/p50*^−/−^ (*n* = 5), *N3tg* (*n* = 5), *p50*^−/−^ (*n* = 3), and *wt* (*n* = 5) mice. ns, not significant; *P ≤ 0.05, **P ≤ 0.01, ***P ≤ 0.001, and ****P ≤ 0.0001 represent significant differences between the indicated groups.

Notch signaling deregulation in T-cell compartment correlates with the expansion of Tregs during T-ALL development in *N3tg* mice (37), depending in part on the activation of canonical NF-κB pathway (38). Besides, canonical NF-κB subunits, such as c-rel and p65, have been described as crucial in Treg development, function, and homeostasis, including in the context of cancer (49–52). Moreover, c-rel has a crucial role in promoting the formation of an enhanceosome specific for the *FoxP3* promoter and that also includes p65, whereas p50 does not activate FoxP3 promoter (52). Notably, a major role of NF-κB1 in Treg biology has been excluded (49, 52, 53), though p50 modulation seems to exert detrimental effects on Tregs in particular conditions, such as in p50/c-Rel double *knock-out* mice (53, 54) or in mice carrying a mutation of p105 (the p50 precursor) that blocks its degradation (55). In this context, *N3tg/p50*^−/−^ model allowed us to verify if NF-κB1 may cooperate with Notch3 in regulating Treg behavior in T-ALL environment. To this end, we examined CD4^+^CD8^−^Foxp3^+^ Treg distributions in the thymus and spleen of *N3tg/p50*^−/−^ mice, by flow cytometry. In the thymus, percentages of Tregs were normal, relatively to those of controls (Figures 8A,B), while their absolute numbers declined (Figure 8C). This event translated in the spleen of *N3tg/p50*^−/−^ mice, where Tregs diminished significantly in percentages (Figures 8D, E), as well as in absolute numbers (Figure 8F). Notably, Treg subset was exclusively reduced when NF-κB1 ablation occurred in the *N3tg* background, suggesting that Notch and NF-κB1 may co-operate in regulating Treg numbers specifically in the context of T-ALL.

### The Apoptotic Rate of DP T Cells and Tregs From *N3tg* Mice Is Enhanced in the Absence of NF-κB1

**Figure 9:**
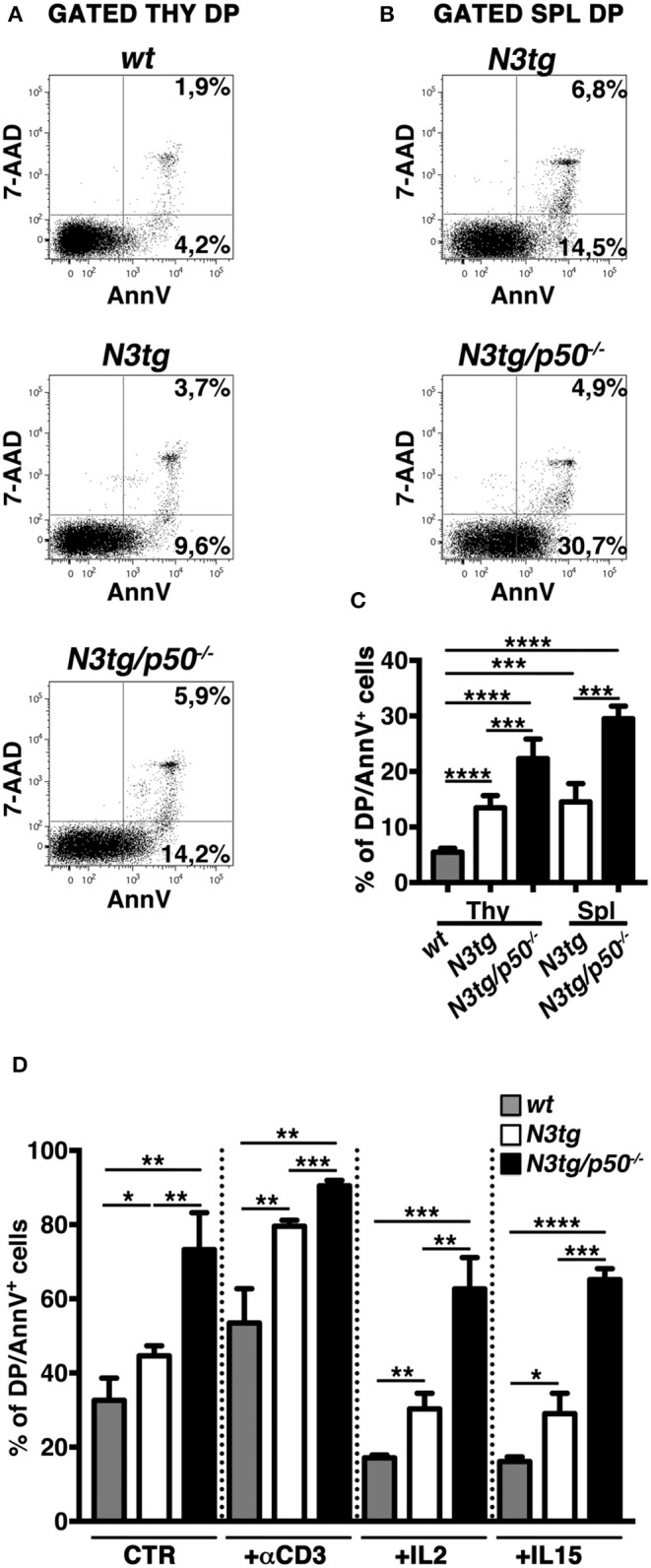
Increased apoptotic rate of CD4^+^CD8^+^ (DP) T cells from *N3tg/p50*^−/−^ mice. Representative dot plots of 7AAD/Annexin V distributions inside gated CD4^+^CD8^+^ (DP) T cells from thymus **(A)** and spleen **(B)** of *N3tg/p50*^−/−^ vs. *N3tg* mice at 4–5 weeks of age. DP thymocytes from *wt* littermates were used as a control. Percentages of both 7 AAD^−^Annexin V^+^ early apoptotic cells and 7AAD^+^Annexin V^+^ late apoptotic cells are indicated inside each cytogram. **(C)** The graph illustrates the percentages of Annexin V^+^ cells inside DP T compartment from thymus (Thy) and spleen (Spl) of *N3tg/p50*^−/−^ and *N3tg* mice at 4–5 weeks of age, as well as from thymus of *wt* controls. The values are presented as mean ± SD of four independent experiments (*n* ≥ 4 mice per group). ***P ≤ 0.001 and ****P ≤ 0.0001 represent significant differences between the indicated groups. **(D)** The graph reports the percentages of DP/Annexin V^+^ cells, assessed by flow cytometry, in thymocytes from *N3tg/p50*^−/−^, *N3tg*, and *wt* mice cultured for 48 h in the following conditions: untreated (CTR), upon TCR activation with anti-CD3 stimulation (+αCD3), or in the presence of survival cytokines such as IL-2 (+IL2) or IL-15 (+IL15). The values are presented as mean ± SD of three independent experiments (*n* = 3 mice per group), in triplicates. ns, not significant; *P ≤ 0.05, **P ≤ 0.01, ***P ≤ 0.001, and ****P ≤ 0.0001 represent significant differences between the indicated groups.

In the attempt to explain the reduction of DP T cells and Tregs in *N3tg/p50*^−/−^ mice, we analyzed apoptosis of these populations, by 7AAD/Annexin V assay on freshly isolated cells. CD4^+^CD8^+^ (DP) T subset from *N3tg* mice presented an accumulation of Annexin V^+^ cells in both the thymus (Figures 9A,C) and spleen (Figures 9B,C), in comparison to *wt* DP thymocytes, used as a control. Interestingly, similar results were reported in transgenic mice with an *lck*-driven constitutive activation of Notch1 intracellular domain (56). However, the percentage of apoptotic cells was even more increased in DP T cells from *N3tg/p50*^−/−^ mice (Figure 9), suggesting that Notch and NF-κB1 may cooperate in regulating survival of DP T cells. Then, we analyzed if the increase of apoptotic rate described above may depend on a T cell intrinsic mechanism or whether it is a secondary effect of the myeloproliferation on microenvironment. To this end, we performed *in vitro* experiments to measure the percentages of apoptotic cells in DP thymocytes from *N3tg/p50*^−/−^, *N3tg*, and *wt* mice that were cultured in the following conditions: untreated, upon TCR activation via anti-CD3 stimulation or in the presence of survival cytokines such as IL-2 or IL-15. Interestingly, significant differences in the percentages of Annexin V^+^ cells persisted among the three groups of DP thymocytes (*N3tg/p50*^−/−^ > *N3tg* > *wt*) in all the conditions tested (Figure 9D), in a similar extent to what observed *in vivo*, thus indicating the cell-autonomous nature of this event.

**Figure 10:**
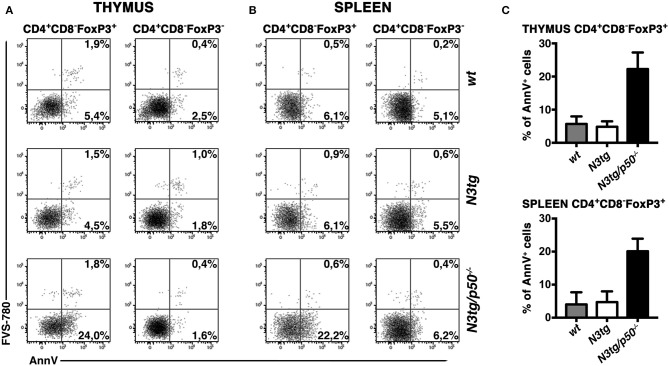
Tregs from *N3tg/p50*^−/−^ double-mutant mice show enhanced apoptosis. Representative dot plots of FVS-780/Annexin V distributions inside gated CD4^+^CD8^−^Foxp3^+^ Tregs or gated CD4^+^CD8^−^Foxp3^−^ cells, from thymus **(A)** and spleen **(B)** of *N3tg/p50*^−/−^*, N3tg*, and *wt* mice at 4–5 weeks of age. Percentages of both FVS-780^−^Annexin V^+^ early apoptotic cells and FVS-780^+^Annexin V^+^ late apoptotic cells are indicated. **(C)** The graph illustrates the percentages of Annexin V^+^ cells inside CD4^+^CD8^−^Foxp3^+^ Tregs from thymus (*upper panel*) and spleen (*lower panel*) of *N3tg/p50*^−/−^*, N3tg*, and *wt* mice at 4–5 weeks of age. The values are presented as mean ± SD of two independent experiments (*n* = 2 mice per group).

Inside Treg subset, the proportion of apoptotic cells was increased in CD4^+^CD8^−^FoxP3^+^ cells from both the thymus (Figure 10A, *left panels* and Figure 10C, *upper panel*) and spleen (Figure 10B, *left panels* and Figure 10C, *lower panel*) of *N3tg/p50*^−/−^ mice. However, percentages of Annexin V^+^ Tregs in *N3tg* mice were comparable to those of *wt* controls, and this apparently rules out a pro-apoptotic role of Notch signaling activation in Tregs. Importantly, no differences were noted in the apoptotic rate of CD4^+^CD8^−^FoxP3^−^ T cells from mice of different genetic backgrounds (Figures 10A,B, *right panels*), used as an internal control. We also excluded defects in the proliferation rates of Tregs that increased in a similar way in both *N3tg* and *N3tg/p50*^−/−^ mice, when compared to those of *wt* counterparts, as measured by Ki67 staining (not shown). In summary, our results suggest that the decrease of DP T and Treg cells in double-mutant mice relies on the enhancement of their apoptotic rate.

### Tregs From *N3tg/p50*^−/−^ Mice Present Altered Responsiveness to IL-2

**Figure 11:**
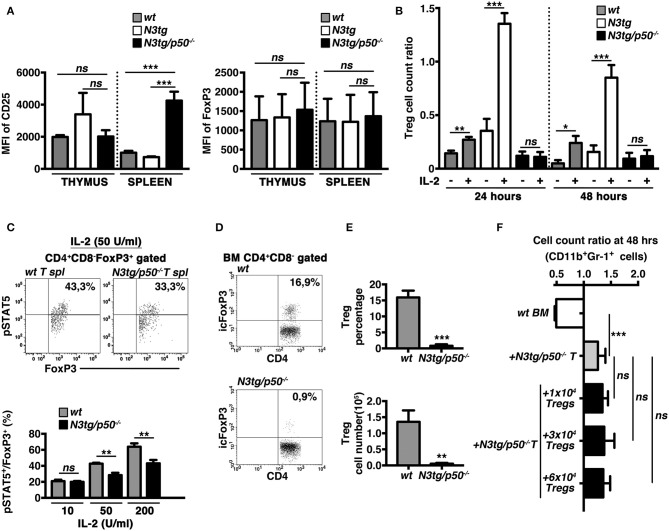
The IL-2/STAT5 signaling is affected in Tregs from *N3tg/p50*^−/−^ mice. **(A)** The histograms represent the MFI of CD25 (*left panel*) and Foxp3 (*right panel*) in gated CD4^+^CD8^−^FoxP3^+^ subsets from thymus and spleen of *wt, N3tg*, and *N3tg/p50*^−/−^ mice at 4–5 weeks of age. **(B)** The graph represents the ratio of the Treg cell count at 24 or 48 h and the related Treg cell count at 0 h in culture samples of T splenocytes from *N3tg/p50*^−/−^*, N3tg*, or *wt* mice at 4–5 weeks of age, in the absence (–) or presence (+) of anti-CD3/IL-2 stimulation. In **(A,B)** the values are presented as mean ± SD of three independent experiments (*n* = 3 mice per group). ns, not significant; *P ≤ 0.05, **P ≤ 0.01, and ***P ≤ 0.001 represent significant differences between the indicated groups. **(C)** In the *upper panels*, representative FACS analysis of pSTAT5 expression inside CD4^+^CD8^−^FoxP3^+^ subset of total T splenocytes (*T spl*) from *N3tg/p50*^−/−^ and *wt* mice at 4–5 weeks of age, upon activation with 50 U/ml of rhIL-2. Numbers inside each cytogram represent the percentages of pSTAT5^+^FoxP3^+^cells inside gated CD4^+^CD8^−^FoxP3^+^ subset. In the *lower panel* the graph represents the percentages of pSTAT5^+^FoxP3^+^cells inside CD4^+^CD8^−^FoxP3^+^ compartment of total T splenocytes from *wt* and *N3tg/p50*^−/−^ mice at 4–5 weeks of age, following the activation with rhIL-2, at the indicated doses. Samples stained without rhIL-2 stimulation served as negative control (not shown). The values are presented as mean ± SD of three independent experiments (*n* = 3 mice per group), in triplicates. ns, not significant; **P ≤ 0.01 represents significant differences between the indicated groups. **(D)** Representative dot plots of icFoxP3/CD4 distributions inside gated CD4^+^CD8^−^ T cells from BM of *N3tg/p50*^−/−^ vs*. wt* mice at 4–5 weeks of age, as assessed by FACS analysis. Percentages of FoxP3^+^CD4^+^ cells are indicated inside each cytogram. **(E)** Mean percentage values (*upper panel*) and absolute numbers (*lower panel*), of Foxp3^+^ Tregs inside the CD4^+^CD8^−^subset in the BM from *N3tg/p50*^−/−^ and *wt* mice, assessed as in **(D)**. The values are presented as mean ± SD of three independent experiments (*n* = 3 mice per group). **P ≤ 0.01, ***P ≤ 0.001 represent significant differences between the *N3tg/p50*^−/−^ samples and the relative *wt* controls. **(F)** The graph represents the ratio of the CD11b^+^Gr1^+^ cell count at 48 h to the related CD11b^+^Gr1^+^ cell count at 0 h in *wt* BM cells cultured alone (*wt BM*), as a basal control, in the co-cultures of *wt* BM cells with T splenocytes from *N3tg/p50*^−/−^ mice (+*N3tg/p50*^−/−^ *T*), as well as in the co-cultures of *wt* BM cells with T splenocytes from *N3tg/p50*^−/−^ mice in the presence of different numbers of CD4^+^CD8^−^EGFP^+^ Tregs purified from the spleen of the Foxp3EGFP “*knock-in*” reporter mice (+*N3tg/p50*^−/−^*T* + 1 × 10^4^/3 × 10^4^/6 × 10^4^ Tregs). Data represent the mean ± SD of three independent experiments (*n* = 3 mice per group), each in triplicates. ns, not significant; ***P ≤ 0.001 represents significant differences between the indicated groups.

The IL-2/IL2R system is extensively involved in regulating many aspects of Treg biology, including the survival. Interestingly, in Tregs from double-mutant mice we did not observe a defective expression on a per cell basis of the CD25 receptor (Figure 11A, *left* panel), or of FoxP3 (as a specific target of the IL-2 signaling in Tregs), (Figure 11A, *right* panel), as evaluated by calculating their Mean Fluorescence Intensity (MFI). Nevertheless, we revealed an altered response to IL-2 of *N3tg/p50*^−/−^ Tregs *in vitro*. Indeed, the Treg cell count ratio increased significantly upon IL-2 stimulation in *wt* samples and even more in *N3tg* samples, with respect to the untreated controls, whereas no differences were noted in IL-2-treated with respect to untreated double-mutant Tregs (Figure 11B). These data suggest that the reduced number of Tregs in double-mutant mice could be related to the impaired response to IL-2. To corroborate our findings, we examined Tregs for the expression of the activated form of STAT5 (pSTAT5), a critical downstream target of IL-2 signaling in Treg development and function. We stimulated T splenocytes with increasing doses of rhIL-2, and then revealed the expression of pSTAT5, by FACS analysis. Data obtained evidenced that *N3tg/p50*^−/−^ mice present a slight but significant decrease in the proportion of pSTAT5^+^ cells inside the CD4^+^CD8^−^Foxp3^+^ Treg compartment, upon activation with high doses of IL-2 (50 or 200 U/ml; Figure 11C), when compared to *wt* controls. These results suggest the presence of an altered IL-2/STAT5 signaling in double-mutant Tregs.

Recently, it was reported that the lack of Tregs in FoxP3-deficient “scurfy” mice induces indirectly a deregulated myelopoiesis, resembling the myeloproliferative trait of our *N3tg/p50*^−/−^ mice (57). Interestingly, *N3tg/p50*^−/−^ young mice (at 4–5 weeks of age), present a dramatic reduction of Treg percentages (Figures 11D, E, *upper panel*), and absolute numbers (Figure 11E, *lower panel*), in the BM, comparable to what observed in their thymus and spleen (see Figure 8). Thus, we investigated the possible role of the Treg lack inside T cell compartment of *N3tg/p50*^−/−^ mice in promoting the CD11b^+^Gr-1^+^ cell growth observed in our co-culture system. To this aim, we performed *in vitro* co-culture experiments of total *wt* BM cells with T cells purified from the spleen of double-mutant mice at 4–5 weeks of age, in combination with different numbers of FoxP3^+^ Tregs, purified from the spleen of the Foxp3EGFP “knock-in” reporter mice (38). Interestingly, the presence of Tregs in the co-culture does not influence significantly the positive effect of *N3tg/p50*^−/−^ T cells on myeloid cell growth (Figure 11F). Thus, it seems unlikely that the lack of Tregs could represent a crucial event in promoting myeloproliferation of double-mutant mice.

### Apoptosis Induction in DP T Cells From *N3tg/p50*^−/−^ MiceCorrelates With Suppression of the p21^Waf1/Cip1^ Protein Expression

**Figure 12:**
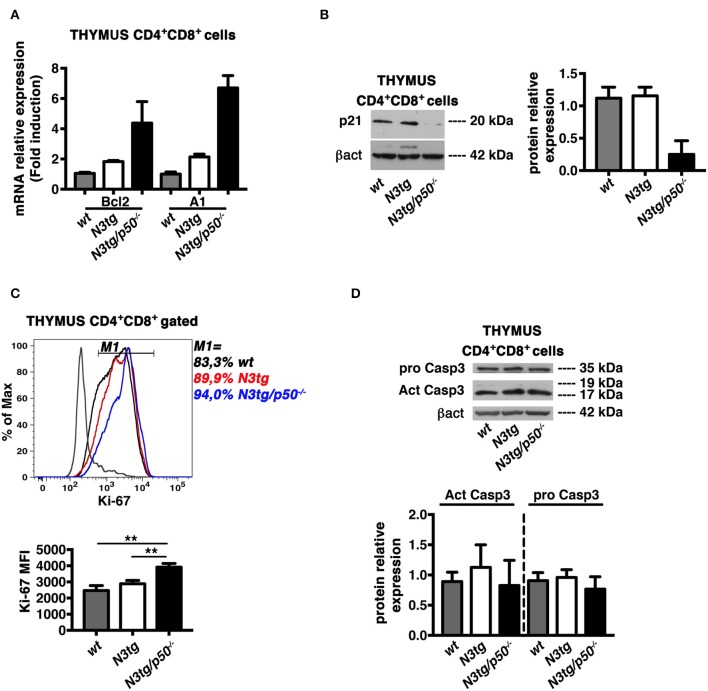
NF-κB1 deletion increases CD4^+^CD8^+^ (DP) T cell apoptosis in *N3tg/p50*^−/−^ vs. *N3tg* mice, through a p21^Waf1/Cip1^-dependent mechanism. **(A)** Relative mRNA expression levels of Bcl-2 and A1 evaluated by real-time RT-PCR in DP T cells isolated from thymus of *N3tg/p50*^−/−^, *N3tg*, or *wt* mice at 4–5 weeks of age. The expression levels of Bcl-2 or A1 in *wt* DP thymocytes were set as 1. Mean values ± SD are shown and they were obtained from two independent experiments (*n* = 2 mice per group), each in triplicate. **(B)** In the *left panel*, representative Western blot analysis of p21^Waf1/Cip1^ protein expression levels in whole-cell extracts of DP T cells isolated from thymus of *N3tg/p50*^−/−^, *N3tg*, or *wt* mice at 4–5 weeks of age. Anti β-actin (β-act) antibody was used as loading control. In the *right panel*, the densitometric analysis of protein expression levels are shown. The values are presented as mean ± SD of two independent experiments (*n* = 2 mice per group). The p21^Waf1/Cip1^ protein expression levels in *wt* DP thymocytes were set as 1. **(C)** In the *upper panel*, histogram of Ki-67 expression inside gated CD4^+^CD8^+^ (DP) T cells from thymus of *N3tg/p50*^−/−^ (blue line), *N3tg* (red line), and *wt* (black line) mice at 4–5 weeks of age, assessed by flow cytometry. The staining negative control is also shown (gray line). M1 gate defines the percentages of Ki-67^+^ cells. Data are representative of three independent experiments. In the *lower panel*, the graph illustrates the MFI of the Ki-67 marker inside CD4^+^CD8^+^ (DP) thymocytes in the same samples as in the *upper panel*. The values are presented as mean ± SD of three independent experiments (*n* = 3 mice per group). **P ≤ 0.01 represent significant differences between the indicated groups. **(D)** In the *upper panel*, representative Western blot analysis of pro-caspase-3 and active caspase-3 protein expression levels in whole-cell extracts of DP T cells isolated from thymus of *N3tg/p50*^−/−^, *N3tg*, or *wt* mice at 4–5 weeks of age. Anti β-actin (βact) antibody was used as loading control. In the *lower panel*, the densitometric analysis of protein expression levels of active caspase-3 and pro-caspase-3 protein are shown. The values are presented as mean ± SD of two independent experiments (*n* = 2 mice per group). In the graphs the protein expression levels in *wt* DP thymocytes were set as 1.

In the attempt to find mechanistic basis for the enhancement of apoptosis in DP T cells from double-mutant mice, we first considered the Bcl-2 protein family, that exerts essential function in cell death regulation. In particular, Bcl-2 and A1 members have antiapoptotic roles and represent important targets of NF-κB in lymphocyte development and hematological malignancies (16, 58). Moreover, the overexpression of Bcl-2 and A1 has been associated to survival of T-lymphoma cells in *N3tg* mice (8). On this premise, we purified DP thymocytes from *N3tg, N3tg/p50*^−/−^, and *wt* mice at 4–5 weeks of age and evaluated the expression of Bcl-2 and A1 mRNAs. Surprisingly, the expression of both Bcl-2 and A1 was increased in samples from *N3tg* DP thymocytes, when compared to *wt* DP thymocytes, and the deletion of NF-κB1 magnified this effect in double-mutant counterparts (Figure 12A). Then, we explored the possibility that NF-κB1 may affect the expression of “cyclin-dependent kinase inhibitor” p21^Waf1/Cip1^. This protein represents a main factor in promoting cell cycle arrest, upon various stimuli. However, p21^Waf1/Cip1^ also acts as an inhibitor of apoptosis in many cell types and through different mechanisms [as reviewed in (59)]. Intriguingly, we detected a downregulation of p21^Waf1/Cip1^ protein expression levels in samples of DP thymocytes from *N3tg/p50*^−/−^ mice (Figure 12B), when compared with both *N3tg* and *wt* samples. We also studied the possible effects of p21^Waf1/Cip1^ downregulation on both the cell cycle and the proliferation of double-mutant DP thymocytes with respect to *N3tg* and *wt* mice counterparts. We did not observe any significant alteration in the distribution of DP T thymocytes of different genotypes in the different phases of the cell cycle, as assessed by 7-AAD staining (not shown), as well as in the percentages of proliferating DP thymocytes, as assessed by the staining with the Ki-67 proliferation marker (Figure 10C, *upper panel*). However, the expression of Ki-67 on a per cell basis was significantly increased in double-mutant DP thymocytes (Figure 12C, *lower panel*). Finally, we revealed that the decrease of the p21^Waf1/Cip1^ protein in double-mutant DP thymocytes does not translate into differences in the protein expression levels of activated cleaved caspase3 or pro-caspase3 (Figure 12D). Overall, our results suggest that NF-κB1 deletion may promote apoptosis in *N3tg* DP T cells through mechanism/s that are dependent on p21^Waf1/Cip1^ expression.

## Discussion

The central role of Notch and NF-κB in the development and progression of T-cell acute lymphoblastic leukemia is well-established (8, 14–18). Nevertheless, little is currently known about combined effects of deregulating these pathways on T-ALL environment. Here, we report that the deletion of NF-κB1/p50 subunit in a murine model of Notch-dependent T-ALL shapes the immunological environment of the disease and influences its outcome. Double-mutant mice show indeed an inhibition of T-cell leukemia progression, evidenced by a strong reduction of pre-leukemic CD4^+^CD8^+^ (DP) T cells in the periphery. At the same time, they develop a dramatic expansion of immature CD11b^+^Gr-1^+^ myeloid cells. Surprisingly, the deletion of NF-κB1/p50 induces the overall effect of reducing survival of *N3tg/p50*^−/−^ mice, with respect to that of *N3tg* animals. It is likely that this effect depends on myeloproliferation. Interestingly, myeloproliferation has already been associated with dysregulation of Notch signaling in hematopoietic system of different murine models, including those of Notch-dependent leukemia (29, 32). Though, Notch has been described as a tumor suppressor in myeloid malignancies [reviewed in (60)]. Together, these observations would support NF-κB as a downstream mediator of Notch signaling in this context.

In order to better characterize the myeloproliferative trait of double-mutant mice, we demonstrate that these mice display an alteration in the myeloid cell development with a significant expansion of the “granulocyte/monocyte progenitor” (GMP) subset in the bone-marrow. Notably, T-cell depletion *in vivo* significantly reduces myeloproliferation in double-mutant mice. Furthermore, T cells from *N3tg/p50*^−/−^ mice are able to improve the growth of CD11b^+^Gr-1^+^ myeloid cells *in vitro*, through a mechanism exerted by *N3tg/p50*^−/−^ DP T cells and that requires cell-to-cell contact. Finally, no ectopic expression of the T cell targeted Notch3-ICN transgene, was observed in the myeloid compartment of *N3tg/p50*^−/−^ mice (PG and AC, unpublished results). Altogether, these results suggest that the myeloproliferation observed in our model relies on non-cell-autonomous processes driven by *N3tg/p50*^−/−^ T cells. However, a more precise definition of the mechanisms involved in this effect deserves additional studies in the future.

NF-κB1 deletion seems to affect immune-environment of *N3tg* mice through a second way, namely the impairment of T cell development. *N3tg/p50*^−/−^ mice present a significant reduction in size of the thymus that reflects the marked decrease in numbers of all thymocyte subsets and mainly of DP T cells. Overall, the enforced expansion of thymic populations that characterize *N3tg* mice vs. *wt* controls [(8) and Figure 6C] is abrogated in *N3tg/p50*^−/−^ mice, thus suggesting a specific involvement of NF-κB1 in mediating this effect of Notch3 dysregulation. The DP T cell compartment is significantly reduced also in the periphery of double-mutant mice, confirming the role of NF-κB1 in sustaining survival of DP T pre-leukemic cells. Instead, NF-κB1 deletion influences marginally the distribution of CD4^+^CD8^−^ and CD4^−^CD8^+^ T cells in the spleen of *N3tg/p50*^−/−^ mice, indicating the presence of compensatory mechanism/s in this process.

There is a third way through which the absence of NF-κB1 may modify T-ALL immune environment, that is represented by the notable reduction of Treg subsets in *N3tg/p50*^−/−^ mice. Importantly, *p50*^−/−^ mice present no major alterations in Treg numbers and function in both the thymus and periphery (49, 52, 53). However, p50 may affect Treg subset under certain conditions (53–55) and its function in Tregs in the context of cancer has not been extensively addressed, so far. To this regard, we revealed the presence of a striking effect of NF-κB1 deletion on Treg survival, that emerges exclusively in the *N3tg* leukemic background. Interestingly, DP thymocytes have been described as the subset in which development of Treg precursors starts (61). Thus, it is possible that the reduction of Tregs that we noted in double-mutant mice depends on the massive reduction of DP thymocytes. The impairment of Treg subsets could also depend on the altered response of *N3tg/p50*^−/−^ Tregs to the IL-2, depending, at least in part, on a reduced activation of STAT5, though the exact mechanisms underlying this effect remain to be elucidated. IL-2 was reported as an essential growth factor for Tregs that is critically required for their homeostasis and metabolic fitness (62). Moreover, NF-κB regulates multiple aspects in the biology of Tregs, including their survival and also their function in tumor environment [as reviewed in (35)]. Thus, it is likely that the deletion of NF-κB1 in the *N3tg* background could influence Treg survival, by inducing alteration of the IL-2 response.

From a mechanistic perspective, our results indicate that the decrease of DP T cells and Tregs in *N3tg/p50*^−/−^ mice arises from an enhancement of apoptotic rate of these subsets. Importantly, the enhancement of apoptosis in T cells from *N3tg/p50*^−/−^ seems to represent an intrinsic event, independent from changes in the microenvironment induced by the expansion of myeloid cells. The apoptosis increase, indeed, persists when double-mutant DP T thymocytes are cultured *in vitro*, also upon TCR activation with anti-CD3 or in the presence of pro-survival cytokines, such as IL-2 or IL-15.

Interestingly, the increase of apoptosis in DP T cells from transgenic mice with dysregulated activation of Notch-ICN was already reported, even if without any definitive explanation (46, 56). Furthermore, it was shown that canonical NF-B pathway activation plays a crucial role in the selection processesof DP thymocytes (13) and promotes their apoptosis (63). Overall, based on our results and literature data, we can speculate that the increased apoptosis of DP T cells from *N3tg* mice, compared to that of *wt* controls, may derive from Notch-dependent constitutive activation of NF-κB. However, Notch activation in thymocytes of *N3tg* mice also promotes other mechanisms (8), that disrupt growth regulation and produce the net result of favoring tumor cell survival. Conversely, the deletion of NF-κB1 in *N3tg/p50*^−/−^ mice can shift the balance toward cell death, possibly through the dramatic decrease of p21^Waf1/Cip1^ protein expression. Many literature data hypothesize that p21^Waf1/Cip1^ protects thymic tumor cells from apoptosis (64) and that NF-κB-dependent induction of p21^Waf1/Cip1^ expression may represent an anti-apoptotic mechanism of resistance in cancer cells, including T-ALL cells (65). Moreover, pharmacological suppression of p21^Waf1/Cip1^ protein by flavopiridol treatment has been experimented on T-ALL Jurkat cells as a successful antileukemic therapy, when combined with HDAC-inhibitors (66). It is noteworthy that the p21^Waf1/Cip1^ protein is involved in the regulation of apoptotic processes that could be dependent or independent from caspase (59, 67). Intriguingly, we reported here that the decrease of p21^Waf1/Cip1^ protein expression in double-mutant DP thymocytes does not impinge on the expression of the active caspase-3 protein. Furthermore, the regulation of apoptosis by p21^Waf1/Cip1^ may occurr independently from its role in the cell cycle (59, 67). To this regard, we observed that the downregulation of p21^Waf1/Cip1^ protein in double-mutant DP thymocytes correlates with an enhanced expression of the Ki-67 proliferation marker on a per cell basis, without any significant alterations of DP thymocyte distribution in the different phases of the cell cycle.

It is important to note that a causative link between Treg reduction and T-ALL progression remains not formally proven in *N3tg/p50*^−/−^ mice. However, Treg accumulation has been highlighted as a negative event in human T-ALL prognosis (36). Hence, it is possible that the inhibition of T-ALL progression observed in *N3tg/p50*^−/−^ mice is not exclusively due to the induction of apoptosis inside pre-leukemic DP T cells, but also depends on the dramatic depletion of Tregs. In this context, we also suggest here that the lack of Tregs does not have a crucial role in driving myeloproliferation of *N3tg/p50*^−/−^ mice, as instead demonstrated in FoxP3-deficient “scurfy” mice (57).

In conclusion, *N3tg/p50*^−/−^ double-mutant mice may represent a novel model to achieve a better understanding of how combined mutations of Notch and NF-κB1 may impact on both the progression of T-ALL and the composition of T-ALL immune-environment in the attempt to identify innovative multiple target therapy for the disease.

## Data Availability Statement

The raw data supporting the conclusions of this article will be made available by the authors, without undue reservation, to any qualified researcher.

## Ethics Statement

The animal study was reviewed and approved by the local Animal welfare committee and was carried out in accordance with the recommendations of the Italian national guidelines for experimental animal care and use and of the European Directive 2010/63/EU.

## Author Contributions

PG and AO designed and performed the experiments, analyzed the data, and wrote the first draft of the paper. NG, CN, and GS performed the experiments. GP analyzed the data. IS critically revised the manuscript. AC supervised the experiments, analyzed the data, and wrote the manuscript.

### Conflict of Interest

The authors declare that the research was conducted in the absence of any commercial or financial relationships that could be construed as a potential conflict of interest.
